# The effect of flywheel training on strength and physical capacities in sporting and healthy populations: An umbrella review

**DOI:** 10.1371/journal.pone.0264375

**Published:** 2022-02-25

**Authors:** Kevin L. de Keijzer, Javier Raya Gonzalez, Marco Beato

**Affiliations:** 1 School of Health and Sports Science, University of Suffolk, Ipswich, United Kingdom; 2 Institute of Health and Wellbeing, University of Suffolk, Ipswich, United Kingdom; 3 Faculty of Health Sciences, Universidad Isabel I, Burgos, Spain; University of Cassino e Lazio Meridionale, ITALY

## Abstract

The aim of this umbrella review was to provide a detailed summary of how flywheel training enhances strength and physical capacities in healthy and athletic populations. The eleven reviews included were analyzed for methodological quality according to the Assessing the Methodological Quality of Systematic Review 2 (AMSTAR 2) and the Grading of Recommendations Assessment, Development and Evaluation (GRADE) criteria. Two were systematic reviews, six were systematic reviews with meta-analyses and three were narrative reviews. Although the included reviews support use of flywheel training with athletic and healthy populations, the umbrella review highlights disparity in methodological quality and over-reporting of studies (38 studies were included overall). Flywheel post-activation performance enhancement protocols can effectively enhance strength and physical capacities acutely with athletes and healthy populations. All relevant reviews support flywheel training as a valid alternative to traditional resistance training for enhancing muscular strength, power, and jump performance with untrained and trained populations alike. Similarly, reviews included report flywheel training enhances change of direction performance—although conclusions are based on a limited number of investigations. However, the reviews investigating the effect of flywheel training on sprint performance highlight some inconsistency in attained improvements with elite athletes (e.g., soccer players). To optimize training outcomes, it is recommended practitioners individualize (*i*.*e*., create inertia-power or inertia-velocity profiles) and periodize flywheel training using the latest guidelines. This umbrella review provides an analysis of the literature’s strengths and limitations, creating a clear scope for future investigations.

## Introduction

Flywheel devices were originally conceived to aid muscle mass maintenance of space travelers exposed to non-gravity environments [1]. More recently, the application of flywheel training as a resistance training method has elicited desirable neuromuscular and task specific adaptations across clinical [2] and sport performance contexts [3–5]. Flywheel devices utilize inertial disc(s) which rotate and store energy during the concentric portion according to the achieved rotational speed, inertial load, and machine characteristics [6, 7]. Subsequently, when the cord rewinds in the eccentric phase, the user is required to resist the rotating disc(s) [4]. The repetitive concentric-eccentric cycles can be performed in a variety of movements, allowing for versatility in training and application [8–10]. The flywheel paradigm is characterized by an unlimited resistance available during the entire range of motion [11, 12], with optimal muscle loading at any given joint angle [2]. If performed appropriately, flywheel exercise may provide a safe, effective, and more demanding eccentric phase (also termed eccentric-overload) than traditional resistance training [2, 4, 13]. Overall, flywheel exercise has been implemented as a valid training methodology to obtain acute and chronic physical capacity and sport performance related adaptations [3, 8, 14, 15].

Flywheel training may be especially beneficial for athletic populations [5, 14]. When applied within post-activation performance enhancement (PAPE) protocols, flywheel methods have shown to be very successful in enhancing strength and physical performance [8, 16–19]. Moreover, flywheel training has reported positive improvements in sport specific capacities after chronic application [20–22]—enhancing jump [23], sprint [24, 25], and change of direction (COD) ability of athletes [26, 27]. Flywheel training has also chronically improved neuromuscular capacity with healthy populations as well as reducing the likelihood of injury or falls and the negative impacts associated with limb disuse and ageing in other populations [2, 28]. With healthy populations, weekly improvements in force production (>2%) and muscle size (±1%) have been reported after only several weeks of training [12, 29–31]. Due to lacking evidence-based guidelines [10], a greater effort has been made towards developing guidelines for monitoring, testing, and periodization of flywheel training [5, 32, 33].

Initial reviews conducted on flywheel training have synthesized research and created practical recommendations and guidelines [4, 9], which provide key references for practitioners and aid decision making—optimizing prescription of training [3]. Yet, inconsistency in the literature exists regarding the effects of flywheel training on physical capacity (*i*.*e*., strength) and sport performance. For example, Maroto-Izquierdo et al. [4] reported a greater magnitude of muscle hypertrophy and physical performance after flywheel training in comparison to traditional resistance training. Conversely, Vicens-Bordas et al. [9] reported no differences between flywheel and traditional resistance training methods for enhancing strength. Several key factors may have influenced the pooled results and conclusions, including the number of databases searched, selection of search syntax, and the data analysis performed [34]. Not only does such inconsistency in the literature pose a challenge to creating definitive recommendations, but it also creates uncertainty regarding direction for future research. Given that some reviews and meta-analyses focus on slightly different aspects of sport performance (*i*.*e*., COD [15], PAPE [8], strength [14, 28]), an overall summary of the impact of flywheel training on physical capacities and strength is needed. Currently, no appropriate analysis or comparison of the quality of evidence supporting the use of flywheel training exists.

A proposed method to reduce the impact of limitations of individual reviews and meta-analyses is to synthesize and appraise them in the form of an umbrella review [34]. Umbrella reviews may help to better understand the evidence landscape by comparing conclusions based on all relevant published data. Umbrella reviews also allow for a greater analysis of bias in the literature which may implicitly affect the validity of the scientific evidence and misguide application [34]. Such analysis, although very important, is generally not performed in reviews and meta-analyses—meaning that bias often infiltrates practice undetected [35]. This umbrella review aims to provide a detailed summary of how flywheel training enhances strength and physical capacities in healthy and athletic populations. The quality and limitations of current evidence (expert-based reviews and meta-analytical evidence) are summarized, and important research avenues are set to explore.

## Methods

### Experimental approach to the problem

The present umbrella review was performed according to current umbrella review guidelines [34] and followed the Preferred Reporting Items for Systematic Reviews and Meta-analysis (PRISMA) statement guidelines [36]. Supporting information can be found in the S1 Checklist.

### Search results

Two reviewers (KDK and MB) conducted a literature search on the following databases: PubMed, SPORTDiscus and Web of Science. The search syntaxes (including keywords and Boolean operators) have been reported here:

Pubmed search: ((((eccentric overload training) OR (flywheel training)) AND (sport performance [MeSH]) OR (muscular strength) Filters applied: Full text, Meta-Analysis, Review, Systematic Review, English; SportDiscus search: ((((eccentric overload training) OR (flywheel training)) AND (sport performance [MeSH]) OR (muscular strength); Web of Science search: TOPIC: (eccentric overload training) OR TOPIC: (flywheel training) AND TOPIC: (sport performance) OR TOPIC: (muscular strength).

ResearchGate was also utilized to find any other relevant texts not identified with the primary literature search. Screening of all bibliographies of selected texts was also performed. Duplicates were identified and removed by two reviewers separately (KDK and MB). The final search was conducted on September 15, 2021. Two reviewers (KDK and MB) independently screened titles and abstracts to identify studies that matched the research aim and inclusion criteria, with a third reviewer (JRG) consulted for discrepancies.

### Inclusion criteria

Search records were limited to full-text articles in English. Utilizing the Participant-Intervention-Comparison-Outcome (PICO) process for evidence-based practice [37], the subsequent inclusion criteria were applied:

1. *Participants*: Ranging from healthy adults and amateurs to professional sporting populations between the ages of 17–40.
2. *Interventions*: Single and multi-component flywheel training programs aiming to enhance physical and/or strength capacity.
3. *Comparison group*: Usual (no additional training) or alternative resistance training.
4. *Outcome measures*: Jumping performance, sprinting performance, change of direction performance, swimming performance, isokinetic strength performance, eccentric hamstring strength performance, one-repetition maximum (1RM) strength, concentric power, eccentric power.

Supporting information and justification for exclusion can be found in S1 File.

### Methodological quality and quality of the evidence assessment

The Assessing the Methodological Quality of Systematic Reviews 2 (AMSTAR 2) checklist was used to determine quality of reviews included [35]. Reviews were classified and scored by two reviewers (KDK and MB), if classification remained unclear, a third reviewer was included in the discussion (JRG). The 16 items of the checklist were answered with a ‘yes’ or ‘no’, with each ‘yes’ equaling 1 point. Reviews were classified as *high* (>80% items satisfied), *moderate* (40 to 80% items satisfied), or *low* quality (<40% items satisfied). An adapted form of the GRADE principles are applied to assess the quality of the evidence provided in the reviews included, as performed previously [38]. Reviews were classified into five GRADE categories: *high*; *moderate*; *low*; *very low*; *no evidence from systematic review*. A review was categorized as *high* quality if it consisted of at least two high-quality studies. Reviews with at least one *high* or two *moderate* quality studies were rated as *moderate* quality. If a review only includes *moderate* quality primary studies and/or primary studies presenting inconsistent results, that review was classified as *low* quality. Reviews are categorized as *very low* quality if they lack *medium* to *high* quality studies. Lastly, if the quality of the primary studies was not assessed by the reviewers, the GRADE system was not applied, and the review was classified as ‘*no evidence from systematic review*’.

### Study coding and data extraction

The following moderator variables were extracted from the included reviews: (1) author details and year of publication, (2) main variables analyzed, (3) main objective of the review, (4) type of investigation, (5) review content (investigations and participants included as well as investigation duration), (6) main findings or conclusions reported. Data extraction, methodological quality assessment and quality of the evidence evaluation were performed independently by two authors (KDK and MB) and discrepancies between the authors were resolved in consultation with a third reviewer (JRG).

## Results

### Search results

**Fig 1:**
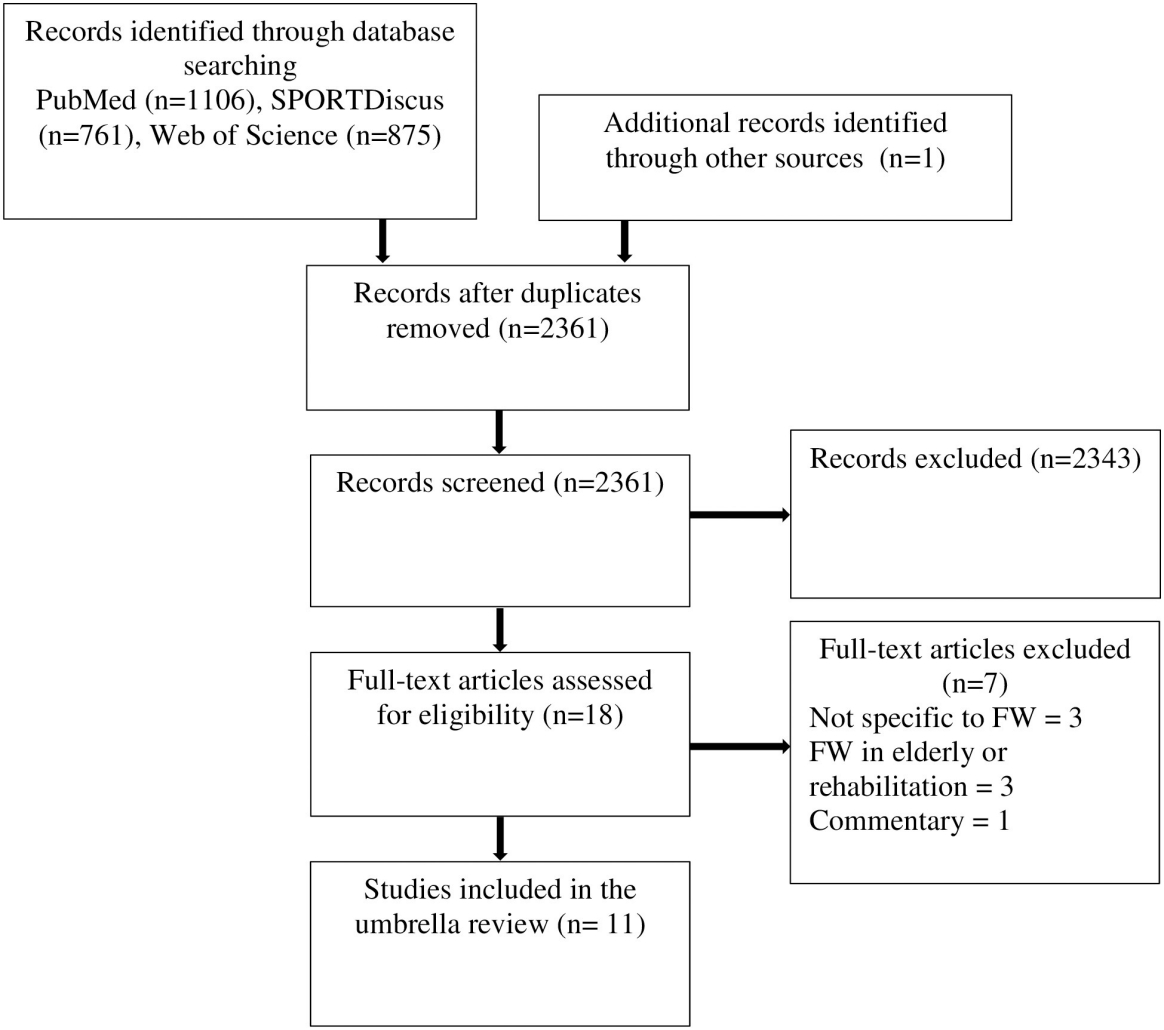
Flow diagram of the study retrieval process.

The flow diagram (Fig 1) shows the retrieval process followed for this umbrella review. Initially, 2742 reviews were identified with the search criteria, while 1 additional review was found through a secondary search. Following this step, duplicate records were removed, and reviews were excluded based on their titles and/or abstracts. 18 full-text reviews were assessed, with 11 reviews included in the umbrella review.

### Descriptive characteristics of the umbrella review

All of the studies that were included in the umbrella review are summarized in Table 1. These reviews were published between 2000 and 2021 and comprised of 38 primary studies corresponding to 608 participants in the experimental groups and 477 participants in the control groups. The 11 selected reviews either analyzed strength, chronic/acute physical capacity, or both. Studies that did not match the inclusion criteria were excluded. Specifically, key information for the reviews included can be found in Table 1. Two included reviews were systematic reviews [28, 39], six were systematic reviews with meta-analyses [4, 9, 14, 15, 40, 41], while three were narrative reviews [2, 8, 13].

**Table 1:** Summary of reviews that investigated the effects of flywheel training on physical capacity and strength.

Authors	Variable	Aim	Type of Investigation	Studies (participants)	Interventions’ duration	Findings/conclusions
Allen et al. (2021)	Sport performance and strength	To synthesize all the flywheel literature specific to male soccer and critically analyze the literature.	Systematic review	9 (119)	6–27 weeks	A variety of training interventions effectively enhance strength, power, jump, and COD measures in male soccer players of varying levels. Uncertainty remains regarding the efficacy of flywheel training for enhancing acceleration and sprint capacity. Certain aspects of training protocols (*i*.*e*., inertia used) were not always clearly reported and must be done in future investigations. Future studies into the effect of flywheel training on elite male and female soccer players are necessary.
Beato et al. (2020)	Post-activation performance enhancement	To summarize the evidence for potentiation protocols utilizing flywheel devices.	Brief review	7 (110)	0.5–12 minutes	A broad range of inertias and rest periods may be utilized to acutely enhance sport performance, with individualization of modulating factors possibly optimizing outcomes. Preliminary methodological guidelines for acutely enhancing athletic performance using flywheel ergometers are presented, with both theoretical rationale and underpinning mechanisms favoring enhanced performance described. Investigations enrolling professional senior team-sport or female athletes are necessary.
Nuñez et al. (2017)	Muscle volume and force	To determine the effects of chronic flywheel training on muscle force and volume of healthy populations.	Systematic review and meta-analysis	7 (113)	4–6 weeks	Flywheel training increases muscle hypertrophy and force in short training periods. An athlete’s training experience facilitates ability to obtain an eccentric overload. Although an eccentric overload does not seem essential for increasing muscle mass, significantly higher improvements in force were seen when eccentric overload was achieved.
Liu et al. (2020)	Change of direction (COD) performance	To determine the effects of eccentric overload training on COD outcomes.	Systematic review and meta-analysis	7 (141)	5–12 weeks	Flywheel training enhances COD performance, providing support for its application in team-based sports. Flywheel training improves muscle activation and braking impulse for tasks that require quick COD. Future investigations should utilize randomized experimental designs, recruiting from professional team sports. Additionally, investigation of how flywheel training impacts various COD tasks (different angles/distances) is of interest.
Maroto-Izquierdo et al. (2017)	Strength, power and sport performance	To investigate the effects of flywheel training on muscle size and capacity in athletic and healthy populations.	Systematic review and meta-analysis	12 (176)	4–10 weeks	The high intensity and brief eccentric overload achieved with flywheel devices are associated with greater improvements in jump height, running speed, CON and ECC force, muscle power and hypertrophy in comparison to traditional resistance training with healthy and well-trained populations. Achieving high speeds and using light moments of inertia during flywheel training appear most effective for inducing muscle adaptations.
Petré et al. (2018)	Strength, power, and sport performance	To analyze the effects of exercise order on muscular hypertrophy.	Systematic review and meta-analysis	14 (235)	4–24 weeks	Flywheel training allows for variable resistance and eccentric overload that differs from traditional resistance training. Both flywheel and traditional resistance training are considered effective for improving strength and hypertrophy in untrained and moderately trained populations. Flywheel training elicits greater strength improvements in well-trained and younger populations than traditional resistance training. Overall, flywheel training seems to be an effective method to improve strength and sport-specific tasks. Real-time feedback during flywheel training may also help to guide intensity and volume prescription.
Raya González et al. (2020a)	Sport performance	To highlight application of flywheel training with team sport athletes.	Narrative review	15 (261)	6–27 weeks	Weekly or bi-weekly flywheel training improves physical performance outcomes. Training load accumulated should be taken into account when implementing flywheel training during in-season periods. Attention should also be given to progressively and individually programming flywheel training after a thorough familiarization period.
Raya-González et al. (2021)	Sport performance	To compare the effect of FW on sports performance of team sport populations.	Systematic review and meta-analysis	9 (172)	5–24 weeks	Flywheel training is an effective tool for enhancing jumping, sprinting, and especially COD performance in healthy active and competitive athlete populations in relatively short periods of time (5–10 weeks). Flywheel ergometers allow for multi-planar and more task-specific movements than traditional resistance training. The characteristics of training applied (volume, inertia and exercise used) should be clearly specified in future investigations.
Raya-González et al. (2021b)	Strength and physical performance	To determine the effect of FW on physical capacity parameters of young and old healthy females.	Systematic review	5 (74)	5–24 weeks	Flywheel training is a potent and time-effective strategy that can safely enhance strength and desirable physical outcomes for young females. Since only knee extension and squat protocols have been investigated, future investigations should determine the effects of different exercises (*i*.*e*., deadlifts, lunges) on strength and performance outcomes with female populations. Furthermore, future studies only investigating females and reporting key factors (*i*.*e*., loading parameters, menstrual cycle profiles) are needed.
Tesch et al. (2017)	Strength and physical performance	To offer perspectives, recommendations, and application within clinical contexts.	Narrative review	11 (171)	5–24 weeks	The enhancement of strength and power seen with flywheel training are underpinned by changes in both muscular hypertrophy and neural activity. Chronic flywheel training may elicit earlier and more robust adaptations than traditional resistance training. Further research into the effects of exercise frequency, volume, and inertia on key outcomes may optimize safety and application of flywheel training.
Vicens Bordas et al. (2018)	Strength and power performance	To determine the effectiveness of flywheel training in comparison to traditional resistance training for enhancing strength and other muscular adaptations.	Systematic review and meta-analysis	4 (36)	6 weeks	The limited evidence available suggests flywheel training is not superior to traditional resistance training for improving muscle strength. Current short-duration interventions may also poorly reflect how long-term flywheel resistance training protocols impact strength outcomes. Further investigation into how loading protocols (*i*.*e*., inertia) and exercise technique impact training outcomes is necessary. Additionally, high quality RCTs (involving randomization, participant blinding) are required to draw firm conclusions for the effect of flywheel training on strength and power.

Abbreviations: 1RM = one repetition maximum; RCT = Randomized control trial; COD = Change of direction; CON = Concentric; ECC = Eccentric.

### Methodological quality assessment and quality of the evidence evaluation

The methodological quality of the 11 included reviews is presented in Tables 2 and 3. Two reviews were rated as *high* quality [9, 15], while six were considered *moderate* quality [4, 14, 28, 39–41] and three of *low* quality [2, 8, 13] using the AMSTAR 2 checklist. Critically, several AMSTAR 2 criteria were not met by a majority of reviews included [35]. Most reviews did not explicitly state that methods were established a priori (item 2). Reviews did not list excluded studies/justify exclusion (item 7), while all lacked a risk of bias assessment of individual studies included in their respective reviews (item 9). Furthermore, most reviews included did not consider the likelihood of publication bias (item 15). According to the adapted GRADE principles applied in the present umbrella review, five investigations were rated as *high* quality [4, 15, 28, 39, 40]. One review was rated as *moderate* quality [14], while the other five reviews did not critically appraise the included studies and could therefore not be assigned a GRADE rating [2, 8, 9, 13, 41].

**Table 2:** Overall results of the AMSTAR 2 and GRADE recommendations for systematic reviews and meta-analyses.

Study	1	2	3	4	5	6	7	8	9	10	11	12	13	14	15	16	AMSTAR 2	GRADE
Allen et al. (2021)	Yes	No	Yes	Yes	Yes	Yes	Yes	Yes	Yes	No	n/a	n/a	Yes	Yes	n/a	Yes	Moderate	High
Nuñez et al. (2017)	Yes	No	Yes	Yes	No	Yes	No	Yes	Yes	No	Yes	No	No	No	Yes	No	Moderate	n/a
Liu et al. (2020)	Yes	No	Yes	Yes	Yes	No	Yes	Yes	Yes	No	Yes	Yes	Yes	Yes	Yes	Yes	High	High
Maroto-Izquierdo et al. (2017)	Yes	No	Yes	Yes	Yes	Yes	Yes	Yes	No	No	Yes	No	No	Yes	No	Yes	Moderate	High
Petré et al. (2018)	Yes	No	Yes	Yes	No	Yes	Yes	Yes	No	No	Yes	Yes	No	No	Yes	Yes	Moderate	Moderate
Raya González et al. (2020a)	Yes	Yes	Yes	Yes	Yes	Yes	Yes	Yes	No	No	Yes	No	No	Yes	Yes	No	Moderate	High
Raya-González et al. (2021)	Yes	No	Yes	Yes	Yes	Yes	Yes	Yes	No	No	n/a	n/a	Yes	Yes	n/a	No	Moderate	High
Vicens Bordas et al. (2018)	Yes	Yes	Yes	Yes	Yes	Yes	Yes	Yes	Yes	No	Yes	Yes	Yes	Yes	Yes	Yes	High	n/a

*Notes*: AMSTAR 2 = Assessing the methodological quality of systematic reviews 2; GRADE = Grading of recommendations, assessment, development, and evaluations; n/a = not applied.

**Table 3:** Overall results of the AMSTAR 2 and GRADE recommendations for narrative reviews.

Study	1	2	3	4	5	6	7	8	9	10	11	12	13	14	15	16	AMSTAR 2	GRADE
Beato et al. (2020)	Yes	No	Yes	Yes	No	No	No	Yes	No	No	n/a	n/a	Yes	Yes	n/a	No	Low	n/a
Raya-González et al. (2020b)	No	No	No	No	No	No	No	Yes	No	No	n/a	n/a	No	No	n/a	No	Low	n/a
Tesch et al. (2017)	No	No	No	No	No	No	No	Yes	No	No	n/a	n/a	No	No	n/a	Yes	Low	n/a

*Notes*: AMSTAR 2 = Assessing the methodological quality of systematic reviews 2; GRADE = Grading of recommendations, assessment, development, and evaluations; n/a = not applied.

## Discussion

This umbrella review provides a detailed summary of how flywheel training enhances strength and physical capacities in healthy and athletic populations and summarizes the quality and limitations of current evidence (expert-based reviews and meta-analytical evidence). The 11 included reviews and the 38 primary studies highlight important considerations for the implementation of flywheel training in sports (*i*.*e*., intensity, volume, frequency, modalities). Importantly, key texts within the current literature that significantly impact practice but are narrative reviews [2, 8, 13] were included.

### Post-activation performance enhancement (PAPE)

The phenomenon defined as PAPE involves the enhancement of voluntary athletic performance following an activation activity (*e*.*g*., resistance exercise) [8, 42]. Phosphorylation of myosin regulatory light chains is suggested to be one of the main peripheral mechanisms associated with muscular performance enhancement [43]. Although the peripheral and central mechanisms underpinning PAPE remain debated [42–44], the research and application of flywheel PAPE protocols has seen a substantial increase over the past decade [8]. The present umbrella review reports that flywheel methods may be particularly effective when aiming to prepare for power-based tasks such as jumping and COD performance [8, 33, 45]. Interestingly, *small* acute improvements in isokinetic concentric and eccentric knee flexor strength have also been reported after flywheel PAPE protocols [8]. In support of such findings, a flywheel deadlift and squat PAPE protocol achieved *moderate* enhancements in eccentric isokinetic hamstring strength [16]. It is important to consider that PAPE protocols were traditionally developed to enhance rapid and power-based tasks more so than strength measures, possibly explaining the difference in literature on the topics [8, 46].

Similarly to strength outcomes, only one study in the umbrella review analyzed sprint speed, reporting a 0.7% (*trivial*) change in 20-meter sprint time [8]. In agreement with the review, another study reported no improvement in 5-meter acceleration performance after a similar flywheel half-squat PAPE protocol [47]. Although single set protocols enhanced diving performance and force parameters of a mixed cohort of swimmers [19, 48, 49], it remains unclear if a single set is sufficient to enhance other physical performance parameters [18]. A greater amount of research has been performed on COD and jumping tasks. The review supports the use of 3–4 sets of 6 repetitions of flywheel half-squats at a variety of inertial loads (0.03–0.11 kg·m^2^) and rest periods (3–9 min) to enhance performance of such measures [17, 47, 50–52]. If practitioners are unsure about optimal inertia selection with flywheel methods, an individualized PAPE protocol and minimal recommended rest periods should be employed [8]. Nonetheless, a large amount of variation in response to PAPE protocols has been noted in the literature, highlighting the importance of modulating factors (*i*.*e*., familiarization and experience) [46]. While similarity between the conditioning activity and subsequent athletic task does not appear to be a key factor for PAPE [16, 44, 53], flywheel training experience may be especially important for optimizing protocols and outcomes [8].

The brief review encapsulates seven studies (110 participants) relative to performance of various physical tasks (Table 1). Its aims were to synthesize preliminary literature, create methodological guidelines [8], and develop flywheel PAPE literature [16, 54]. Nonetheless, the conclusions of this review have some limitations considering the AMSTAR 2 checklist. Neither study selection nor data extraction was performed in duplicate, and authors did not justify study exclusion from the review. The authors did not perform a risk of bias assessment nor consider the potential effects of missing such an analysis [35]. Although the present review is valuable [8], future investigations on flywheel PAPE protocols based on high level evidence are necessary [8, 33].

### Chronic performance

#### Strength

The development of muscular strength is paramount to improving key muscular qualities, such as rate of force development [45]. Such improvements play key roles in optimizing sport performance and neuromuscular function of athletic and healthy populations [2, 45]. The interest in investigating the benefits of flywheel training for enhancing strength are highlighted by the multiple systematic and narrative reviews on the topic [2, 9, 13].

*Systematic reviews*. All of the systematic reviews (*moderate* to *high* AMSTAR 2 and *no rating* to *high* GRADE) supported flywheel training for enhancement of strength performance [4, 9, 14, 28, 39, 41]. Most of the reviews conclude that flywheel training is a valid alternative to traditional resistance training [9, 14, 28, 41]. In fact, Petre at al. (2018) reported large improvements in maximal strength (ES = 1.33) when 1–3 sessions were performed per week [14]. Such improvements in strength also appear to occur alongside rapid changes in pennation angle and fascicle length [12, 29]. The reported improvements in strength after flywheel training may be due to the effective development of both peripheral and central mechanisms [14]. Specifically, greater muscle activation, alterations in muscle morphology and to the length-tension relationship may be key to enhancements seen in the literature following flywheel training [12, 29, 55]. Importantly, strength improvements are typically seen after 5 to 10 weeks of flywheel training [4, 9, 14, 39], with one review highlighting that well-trained individuals may benefit more so than untrained individuals when training with the flywheel method [14]. Although it remains unclear why this occurs, it is possible that greater training experience or strength may allow for greater activation and control of the musculature during intense eccentric contractions [56].

Discord between systematic reviews exists regarding flywheel training and whether it is more effective [4] or equivalent to traditional resistance training for enhancing strength [9, 14, 28, 41]. Such differences are most probably due to the difference in inclusion criteria (*i*.*e*., different control groups or tests/measurements) which alter the findings of the meta-analyses and conclusions drawn from the systematic reviews [4, 9]. It remains difficult to conclude whether or not flywheel training is more effective than traditional resistance training and whether it is more effective with well-trained populations. Conclusions are limited by lacking well designed studies directly comparing the two methodologies [9, 30], with future research needed to clarify if differences exist.

*Narrative reviews*. Narrative reviews included in this umbrella review conclude that flywheel training is a valid resistance training method for enhancing strength (*low* quality and no GRADE applied) [2, 13]. Specifically, investigations applying flywheel training in a weekly and bi-weekly manner have enhanced strength during the in-season period with athletic populations [20, 21, 57]. Flywheel training can elicit larger force, torque, and muscle activation during the eccentric phase in comparison to the concentric phase [11, 12, 58]. In support of this, several reviews highlight that such an “eccentric overload” can be particularly beneficial for strength outcomes [2]. The exposure to optimized loading during both concentric and eccentric phases experienced with flywheel training may partly explain the distinct adaptations experienced in such short periods of time [11, 12, 29]. Such improvements are very attractive when training frequency must be reduced [10, 59, 60]. Nonetheless, caution is also warranted with such outcomes largely dependent upon appropriate movement familiarization and technique [2, 13]. Although future investigation into the effects of training experience on strength outcomes is warranted [31], weekly or bi-weekly flywheel training can be considered a viable method to enhance strength with athletes [20, 21, 57, 61]. More recently, interventions individualizing and progressively increasing inertia have been performed—enhancing performance and strength measures similarly to traditional resistance training [61, 62]. Nonetheless, future investigation into the effects of optimal frequency, varying training specificity, individualization, and appropriate progression criteria are necessary to develop application [13].

#### Sprint

Sprint performance is frequently investigated because of its relevance to sporting demands and more generally because of its role as a physical capacity [4, 40]. Nonetheless, no review has been specifically dedicated to solely investigating speed or sprinting ability, as performed with strength [9] or COD performance [15, 28].

*Systematic reviews*. Four systematic reviews [4, 14, 39, 40] (*moderate* or *high* quality) were included in the present umbrella review. Training protocols with varying training exposure elicited favorable adaptations amongst soccer players [21, 62] and team-sport athletes [23–25]. The versatility of flywheel training in team-based environments is supported by the enhancements in sprint performance after squat [63], leg curl [21], leg press [23], and multi-exercise flywheel programs [26]. Favorably, such improvements in performance are seen after 5–10 weeks of training [40]. Furthermore, the application of flywheel multi-planar and sport-specific movements may also be beneficial for enhancement of sprint performance [40]. Changes towards faster muscle phenotypes [64] or changes in rate of production and retention of power during sprint strides are among several justifications for the benefits seen in sprint performance associated with eccentric and flywheel training [14]. The enhancement of concentric and eccentric strength has been seen alongside enhancement of sprint performance in athletic populations [4, 25]. Nonetheless, some investigations have reported limited benefits or *trivial* enhancements in sprint performance after flywheel training [20, 22, 26, 27, 65]. Recently, a randomized control trial investigating the effects of flywheel lateral squat training on physical capacity of U16 elite soccer players reported no enhancement of sprint performance—possibly due to the low training dose used (a weekly session) [27]. Alternatively, differences in familiarization protocols or training experience of the athletes may be key factors impacting outcomes [40]. One review specifically suggests that variation in sprint test outcomes may be specifically related to varying distances and instructions during sprint tests [14]. The literature supports the notion that flywheel training must be appropriately dosed to optimize efficacy [14, 39], which may not be possible in the context of prolonged congested fixture periods in season, when limited training time is available for athletes [59]. Of the four systematic reviews, one had large heterogeneity (I^2^ = 89%) in the variable sprint performance [14] and only one review accounted for the reliability measures of included individual studies [39]. Further investigation of low-dose flywheel training is therefore warranted to determine the effects of manipulating different training parameters during in-season periods on sprint performance [20, 25].

*Narrative reviews*. Flywheel training programs included in the reviews are typically performed weekly or bi-weekly with team sport athletes [2, 13], reflecting flywheel training frequency reported in professional soccer [10]. Although Tesch et al. (2017) [2] reported that sprint performance can be enhanced after application of flywheel training, the other narrative review reported inconsistent findings in both shorter (<10 m) and longer sprints (>20 m) [13]. Although inconsistent findings in sprint performance have been reported after flywheel training, several investigations highlight that other resistance training methods were similarly ineffective for enhancing sprint performance during in-season periods. For example, neither flywheel squat nor 80%1RM squat training enhanced 10 m and 30 m sprint performance with semi-professional soccer players [20]. Similarly, neither plyometric and resistance training protocols nor the addition of flywheel training to such training enhanced sprint performance outcomes with young males [26, 65]. The present review highlights the need for further high-quality studies on the topic (*i*.*e*., randomized controlled trials) to better understand how to optimally implement flywheel training to develop sprint performance.

#### Change of direction (COD)

COD are often characterized by demanding braking actions followed by immediate and high propulsive forces required to re-accelerate in a new direction [66]. Such actions are commonly performed in sport and are predominantly of interest for team-based sport athletes more so than healthy populations [67, 68]. Flywheel devices have been utilized to replicate similar movement patterns and transition from eccentric to concentric phases, which are believed to be particularly beneficial for enhancing change of direction outcomes [69, 70]. Logically, improvements in COD ability are therefore expected after appropriate application of flywheel training with athletic populations.

*Systematic reviews*. Two systematic reviews and meta-analyses investigating this topic were rated *moderate* or *high* on both AMSTAR 2 and GRADE [15, 40]. Although only involving three studies, Raya-Gonzalez et al. (2020) [40] reported flywheel training elicits significantly favorable outcomes in comparison to control conditions amongst athletes. Similarly, Liu et al., (2020) [15] reported beneficial COD outcomes after flywheel training. Such improvements in COD performance with team-based sport athletes were reported in comparison to regular training [66] and to traditional resistance training [20, 23, 26, 71]. Flywheel training appears to improve performance by reducing braking time and enhancing braking impulse during COD movements [15]. A systematic review by Allen et al. (2021) [39] (rated *moderate* and *high* on AMSTAR 2 and GRADE) also supported the efficacy of flywheel training for enhancing COD ability with adult male soccer players. Specifically, a variety of protocols appeared effective for enhancing COD performance parameters with youth and semi-professional soccer players [20, 26, 66]. Even though such improvements are reported, appropriate familiarization and adequate flywheel training technique are key to ensure COD performance enhancement with athletes [39, 40].

*Narrative reviews*. Two narrative reviews (rated *low* on AMSTAR 2, no GRADE applied) reached similar conclusions to the systematic reviews previously mentioned [2, 13]. Specifically, the authors highlight that other practical limitations affect flywheel training frequency and suggest that weekly training may still be effective for obtaining COD adaptations [20, 26]. Similarly, Raya-Gonzalez et al. (2020) [13] propose at least 8–11 weeks (one training session a week) and 6 weeks (two training sessions a week) of flywheel training be performed to enhance COD performance. Individualizing inertia chosen may further enhance COD outcomes [2, 58], although the optimal method to determine appropriate exercise inertia (intensity) remains unclear [6]. Nonetheless, the various tests (L-drill, V-cut) included in the narrative reviews suggest that flywheel training can enhance different types of COD tasks required in team-based sports [2, 13]. Although the recommendations provided by the narrative reviews presented are useful, their methodological limitations should be considered by practitioners (Table 2).

Although the reviews performed on COD performance and flywheel training involve a variety of team-based sports, they are predominantly based on a limited amount of investigations [13, 15, 39, 40]. This reflects the smaller number of investigations assessing the effects of flywheel training programs on COD ability in comparison to other physical qualities (*e*.*g*., jump performance). The obtained enhancements of jump ability in athletic and healthy populations also seem to be more consistent when compared to COD outcomes, which may be explained by a greater variation and disparity in training doses and tests utilized.

#### Jump

Jumping performance is often utilized as a key indicator for lower-limb power, strength and physical ability with both healthy and athletic populations [4, 72]. Improvements in energy production and storage during the stretch-shortening cycle may be related to the transition from eccentric to concentric phases during flywheel training [73]. Moreover, the high eccentric demands of flywheel exercise may be an effective method to stimulate lower limb strength and power parameters, which can have a positive transfer to jumping performance [40].

*Systematic reviews*. Four systematic reviews and meta-analyses have specifically investigated the impact of flywheel training on jumping performance [4, 14, 39, 40]. The first systematic review on the topic (rated *moderate* and *high* on AMSTAR 2 and GRADE, respectively) conducted by Maroto-Izquierdo et al., 2017 [4] reported significant improvements (p < 0.01) in jump ability after 4–10 weeks of flywheel training, although it only involved 3 studies. Petré et al. [14] and Raya-González et al. [40] meta-analyzed 7 and 8 studies, respectively. The greater number of studies included, and the quality of the reviews (rated as *moderate* and *high*) further enhances confidence in application of flywheel training for jump performance enhancement in both athletic and healthy populations. In agreement with previous findings [4], both reported enhancement of jump performance after flywheel training protocols spanning 5–24 weeks [14, 40]. Similarly, another systematic review investigating the effects of flywheel training on young female populations reported improvements in jump ability [28]. The systematic review focused on female populations reported a greater effect when participant level was lower (healthy adults vs. team-sport athletes) and when weekly frequency was increased (1 vs. 3 weekly sessions) [28]. The systematic review by Allen et al. (2021) [39] observed that flywheel training frequently enhanced jumping ability of soccer players (only 1 of 7 studies did not report improvements). Overall, despite the promising results of the aforementioned findings, the meta-analyses included a variety of participants (healthy adults and team-sport athletes) [14, 40]. In fact, one of the meta-analyses included also reported high heterogeneity (I^2^ = 81%) [14], limiting conclusions. Further investigation into how flywheel training can enhance jumping performance of athletic populations may help optimize practical recommendations and conclusions.

*Narrative reviews*. Two narrative reviews and commentaries discussed the application of flywheel training for jump performance enhancement [2, 13]. The narrative review by Tesch et al. [2] reported enhancement of jump ability in healthy populations after flywheel training but does not provide conclusions for healthy athletic populations. On the other hand, Raya-González et al. [13] reviewed the application of how the flywheel paradigm is used to enhance jumping performance specifically in team-sport athletes. This review reported 3–10% improvements in countermovement jump performance when 4–6 sets of 6–10 repetitions of all-out flywheel half-squats were performed [13]. Nonetheless, when multi-exercise programs (including flywheel training) were implemented, no significant improvements in jump ability were seen [13]. Differences in response to training highlight that training specificity and exercise selection may be important considerations when designing flywheel training programs. When implementing flywheel training, it is recommended that practitioners use lower inertias for power-based actions and individualize training (*i*.*e*. create inertia-velocity or inertia-power profiles) if feasible [6, 32].

*Limitations and future directions*. A limitation of the present umbrella review is that majority of the reviews included utilized the same primary studies, highlighting a considerable over-reporting among reviews. As addressed earlier in the umbrella review, several limitations related to the methodological quality of the systematic and narrative reviews included affect the conclusions on the efficacy of flywheel training for developing strength and physical capacity. To enhance future investigations, authors are recommended to report all loading parameters (*i*.*e*., inertia) utilized in their protocols to enhance application and reproducibility. Furthermore, it is recommended to avoid using the term “eccentric overload” to define flywheel training. Instead, the term “eccentric overload” should only be used when confirmed (with appropriate measurements, e.g., encoder) and should be defined as a larger eccentric output in comparison to the respective concentric output—in line with previous recommendations [2, 33]. The present review echoes the need for further high-quality studies on PAPE and chronic flywheel protocols with elite athlete and female populations to enhance application [3, 28]. Research into the differences between flywheel and traditional resistance training for strength and physical capacity parameters is of interest and necessitates specific attention [3, 9]. Finally, further investigation into loading parameters, training frequency, and familiarization will enhance the quality of training protocols and outcomes.

## Conclusions

This umbrella review provides a detailed summary on the effects of flywheel training for strength and physical capacity parameters in healthy and athletic populations and summarizes the quality and limitations of current evidence. Moreover, it provides an analysis of the literature’s strengths and limitations, creating a clear scope for future investigations and reviews. The 11 included reviews (including 38 primary studies) highlight that application of flywheel training with sports and healthy populations varies in prescription of exercise intensity, volume, frequency, and exercises. The variation in populations, protocols utilized, and methodological quality ratings of reviews included should be individually considered when interpreting findings. The evidence on flywheel PAPE protocols highlights that such protocols are effective for enhancing isokinetic hamstring strength, jump, and COD performance with athletes, although further high-quality investigations are necessary to confirm current findings. All reviews support use of flywheel training for enhancing muscular strength, power, and jump performance with healthy and athletic populations. All systematic and narrative reviews also conclude flywheel training improves change of direction performance—although conclusions are limited to fewer investigations than the aforementioned parameters. The reviews investigating the effect of flywheel training on sprint performance report some inconsistency in attained improvements with elite athletes (e.g., soccer players). To optimize training outcomes, it is recommended practitioners individualize (*i*.*e*., create inertia-power or inertia-velocity profiles) and periodize flywheel training using the latest guidelines [5, 32].

## Supporting information

S1 File Excluded studies (with justification). (DOCX)

S1 Checklist PRISMA 2020 checklist. (DOCX)
