# Supplementary material for: The effect of flywheel training on strength and physical capacities in sporting and healthy populations: An umbrella review
Source: PLoS One. 2022 Feb 25;17(2):e0264375. doi: 10.1371/journal.pone.0264375 (PMC8880830; doi:10.1371/journal.pone.0264375)
Supplement: S1 File — (DOCX) [file pone.0264375.s001.docx]

**Excluded Studies (with justification)**

**Allen (2021)**

Fiorilli et al (2020) (13 yrs old)

Gonzalo-Skok et al., (2019) (15 yrs old)

Raya Gonzalez (2021) (age not reported)

**Javier Nuñez (2017)**

Onambele 2008 (70 yrs old)

Naczk 2014 (28) (Physically active but type of exercise not relative to team-based sport demands - Shoulder adduction on ITMS)

**Liu et al 2020**

Fiorilli et al (2020) (13 yrs old)

Sanchez-Sanchez 2019 (Combined high intensity interval training and flywheel training)

Chaabene 2019 (Nordics, no FW)

Siddle 2019 (Nordics, no FW)

Bourgeois 2017 (15 yrs old; No FW)

Lockie 2014 (Enforced deceleration program, no FW)

**Maroto-Izquierdo et al. (2017)**

Naczk 2014 (Shoulder adduction/abduction exercises)

Naczk 2016 (Elbow flexor/extensor exercises)

**Petré et al. (2018)**

Bruseghini 2015 (68 yrs old)

Caruso 2005 (59 yrs old)

Lundberg 2012 (Aerobic exercise + FW vs. FW)

Lundberg 2014 (Aerobic exercise + FW vs. FW)

Naczk 2014 (Should adductor/abductor exercises)

Naczk 2016 (Elbow flexor/extensor)

Onambele 2008 (70 yrs old)

Owerkowicz 2016 (Aerobic and FW)

**Raya González et al. (2020)** [1]

Raya Gonzalez 2018 (15 yrs old)

**Raya-González et al. (2021b)** [2]

Onambele 2008 (70 yrs old)

Sanudo 2019 (65 yrs old)

**Tesch 2017**

Alkner, 2003;Alkner Tesch 2004a; Alkner and Tesch, 2004b; Alkner et al., 2016; Rittweger et al., 2005; Nielsen et al., 2016; Irimia et al., 2017; Cotter 2015; Owerkowicz 2016; Tesch 2004b; Haddad 2005; Fernandez Gonzalo 2014a (Concurrent aerobic/FW, space, limb unloading, no sport performance)
Bruseghini 2015 (68 yrs old, no performance measures, HIT then FW)
Onambele 2008 (70 yrs old)
Romero-Rodriguez 2011 (Athletes with tendinopathy)
Abat 2014/2015 – (Patellar tendinopathy)
Greenwood 2007 – (Unilateral knee injury)
Fernandez Gonzalo 2014c (63 yrs old, stroke patients)
Fernandez Gonzalo 2016a (61 yrs old, 14 stroke patients)
Oliviera 2015 (46 yrs old, 24 multiple sclerosis patients)
Sarmiento 2014 (78 yrs old, Alzheimer’s)

**Vicens Bordas 2018**

Greenwood (37±13 yrs. with history of knee injury)

Onambele (69 yrs old)

Caruso 2005 (58 yrs old)

**Excluded Reviews from umbrella review:**

Douglas J, Pearson S, Ross A, McGuigan M. Eccentric exercise: Physiological characteristics and acute responses. Sports Medicine. 2017. doi:10.1007/s40279-016-0624-8

(Not specific to FW)

Kingma, J. J., de Knikker, R., Wittink, H. M., & Takken, T. (2007). Eccentric overload training in patients with chronic Achilles tendinopathy: a systematic review. *British journal of sports medicine*, *41*(6), e3-e3.

(Injured population)

Tinwala, F., Cronin, J., Haemmerle, E., & Ross, A. (2017). Eccentric strength training: A review of the available technology. *Strength and Conditioning Journal*, *39*(1), 32-47.

(Reviewing technology)

Vogt M, Hoppeler HH. Eccentric exercise: Mechanisms and effects when used as training regime or training adjunct. Journal of Applied Physiology. 2014. doi:10.1152/japplphysiol.00146.2013

(Not specific to FW)

Wonders J. Flywheel training in musculoskeletal rehabilitation: A clinical commentary. Int J

Sports Phys Ther. 2019;14: 9941000.Available:http://www.ncbi.nlm.nih.gov/pubmed/31803531

(Rehabilitation)

Mosteiro-Muñoz, F., & Domínguez, R. (2017). Effects of inertial overload resistance training on muscle function. *Revista Internacional de Medicina y Ciencias de la Actividad Física y del Deporte*, *17*(68).

(Rehabilitation)

Beato M, Dello Iacono A. Implementing flywheel (isoinertial) exercise in strength training: current evidence, practical recommendations, and future directions. Front Physiol. 2020;11. doi:10.3389/fphys.2020.00569

(Commentary)
